# A multi-proxy approach to reconstruct chronology, human mobility, and funerary practices at the Late Bronze-Early Iron Age urnfield of San Valentino (San Vito al Tagliamento, Italy)

**DOI:** 10.1371/journal.pone.0309649

**Published:** 2024-11-07

**Authors:** Giacomo Capuzzo, Elisavet Stamataki, Michael Allen Beck De Lotto, Silvia Pettarin, Philippe Claeys, Nadine Mattielli, Giovanni Tasca, Christophe Snoeck

**Affiliations:** 1 Bagolini Laboratory: Archaeology, Archaeometry, Photography (LaBAAF), Department of Humanities, University of Trento, Trento, Italy; 2 Research Unit: Anthropology and Human Genetics, Department of Biology of Organisms and Ecology, Université Libre de Bruxelles (ULB), Brussels, Belgium; 3 Laboratoire G-Time, Department of Geosciences Environment and Society, Université Libre de Bruxelles (ULB), Brussels, Belgium; 4 Archaeology, Environmental Changes & Geo-Chemistry, Vrije Universiteit Brussel, Brussels, Belgium; 5 Department of Cardiac, Thoracic, Vascular Sciences and Public Health, University of Padua, Padua, Italy; 6 Museo Civico “Federico De Rocco”, San Vito al Tagliamento (PN), Italy; Austrian Academy of Sciences, AUSTRIA

## Abstract

The site of San Valentino in San Vito al Tagliamento is one of the main urnfield cemeteries in northeastern Italy. Archaeological excavations carried out in the seventies brought to light a cremation cemetery consisting of mainly urn graves with pottery and metal artefacts as grave goods. These materials suggest that the individuals buried in San Valentino were not an isolated local community but had intense contacts with other north-Adriatic communities, in particular with the neighbouring Veneto area, as suggested by the close similarity of the biconical vessels with those recovered in the graves of Este. This paper provides the first osteological study of a preserved sample of individuals buried at San Valentino and uses an innovative multi-proxy approach to refine the chronology of the site through radiocarbon dating of bone apatite, investigate human mobility using strontium isotopes on calcined human remains, and reconstruct the funerary practices by combining FTIR-ATR data with carbon and oxygen isotope ratios on cremated bones. The results date the cemetery to the end of the Late Bronze Age and the Early Iron Age, with a sporadic occupation in the fourth century BC. Strontium isotopes and concentrations show the analysed individuals buried at San Valentino were a local community that exploited nearby food resources. Interestingly, variations in cremation conditions were detected between San Valentino and the contemporary sites of Velzeke, Blicquy, Grand Bois, and Herstal, located in Belgium, by using FTIR-ATR and carbon and oxygen isotope data. This multi-proxy approach applied to the study of cremated human remains can open new research possibilities, being potentially extendable to the study of many pre- and proto-historic and historic communities that practised cremation.

## Introduction

The last two decades have been characterised by a flourishing number of studies on cremated bones from archaeological contexts. Scientific advances in methods and techniques have opened new horizons in the study of past populations that practised cremation. In 1998, it was demonstrated that calcined bone can be radiocarbon (^14^C) dated using the carbon present in the inorganic fraction of bone commonly called bioapatite or bone apatite [1, 2], allowing to obtain new chronological data from cremation burials that were previously dated only by the typology of grave goods associated with the cremated remains or using charcoal. More recently, it was also shown that calcined bone (totally white) provides a reliable substrate for strontium isotope analyses [3]. Consequently, cremated human remains have started to be used as a proxy to study past population dynamics such as variations in palaeomobility and landscape use [4–11]. Recent studies have shown that the combined use of Fourier Transform Infrared Spectroscopy (FTIR), and carbon and oxygen isotope analysis on bone apatite can provide new knowledge regarding the burning conditions in the past (temperature, type and/or amount of fuel, pyre structure and location, body position, extinguishing methods) [12–15]. Eventually, it has been proved that strontium isotopes (^87^Sr/^86^Sr) and concentration ([Sr]) analyses of cremated human remains can also be used to infer changes in food consumption patterns [16, 17]. In concomitance with these scientific advances, new methods for sexing and ageing cremated remains have also been developed [18–20].

The aim of this paper is to implement state-of-the-art analytical techniques in the analysis of cremated human remains to refine the chronological framework and to investigate human mobility, and the way cremation was performed in one of the main Early Iron Age (EIA: 9^th^-5^th^ centuries BC) urnfields in northeastern Italy, the cemetery of San Valentino. The Urnfield culture encompasses a diverse array of local cultures across Europe during the Late Bronze Age (LBA) and the Early Iron Age (EIA) in the second half of the 2^nd^ and the early 1^st^ millennium BC. This cultural phenomenon is defined by a shared funerary practice involving cremation deposits placed within ceramic urns [21, 22].

**Fig 1:**
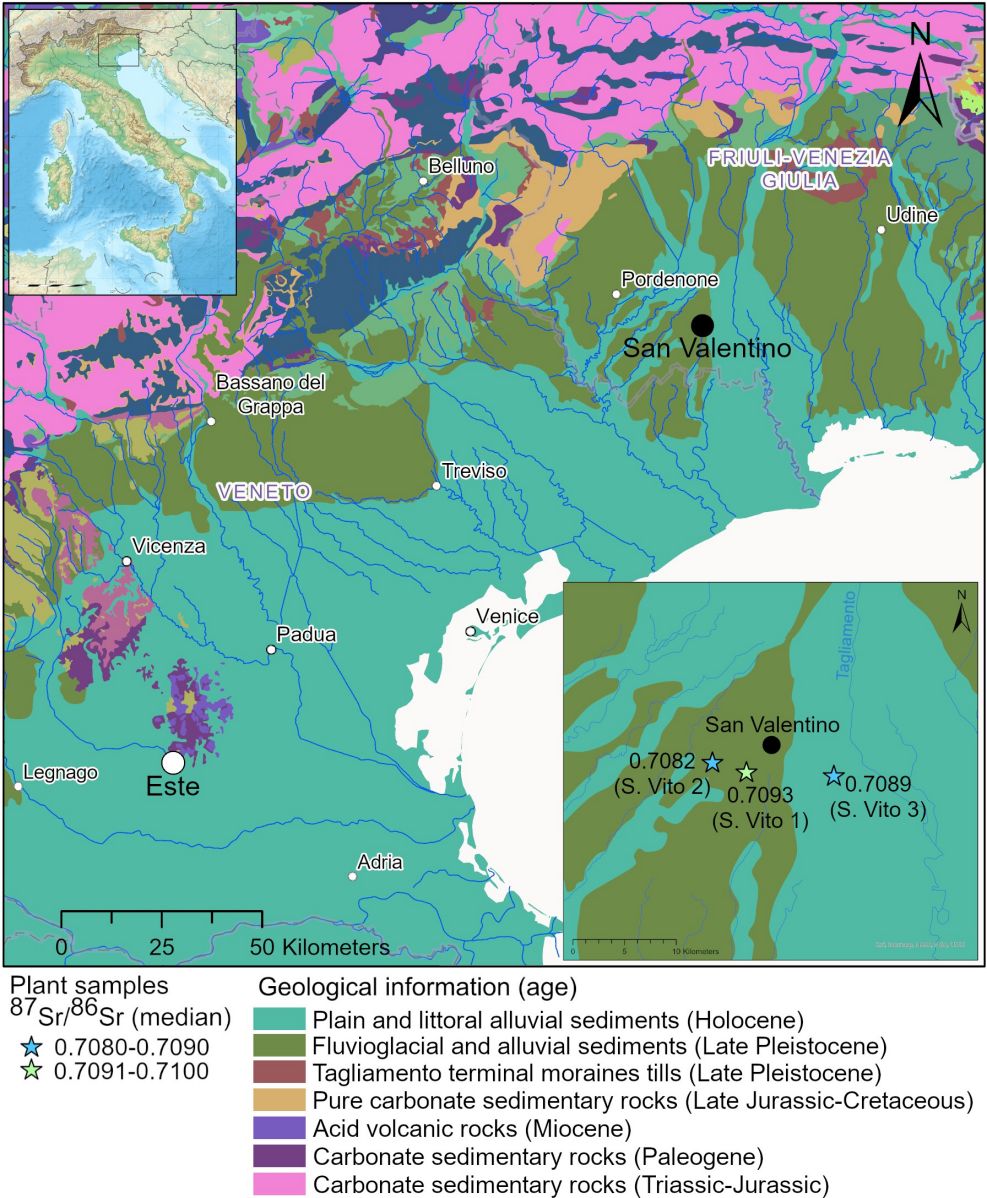
Location of San Valentino and Este sites on the global GLiM map [31] integrated with information from the geological map of the Friuli Venezia Giulia [32]. Median values for bioavailable ^87^Sr/^86^Sr measured in plant samples collected around San Valentino are indicated in the map, created using ArcGIS Pro. Blank physical map of Italy from Eric Gaba (Wikimedia Commons user: Sting) and Wikimedia Commons user: NordNordWest.

**Fig 2:**
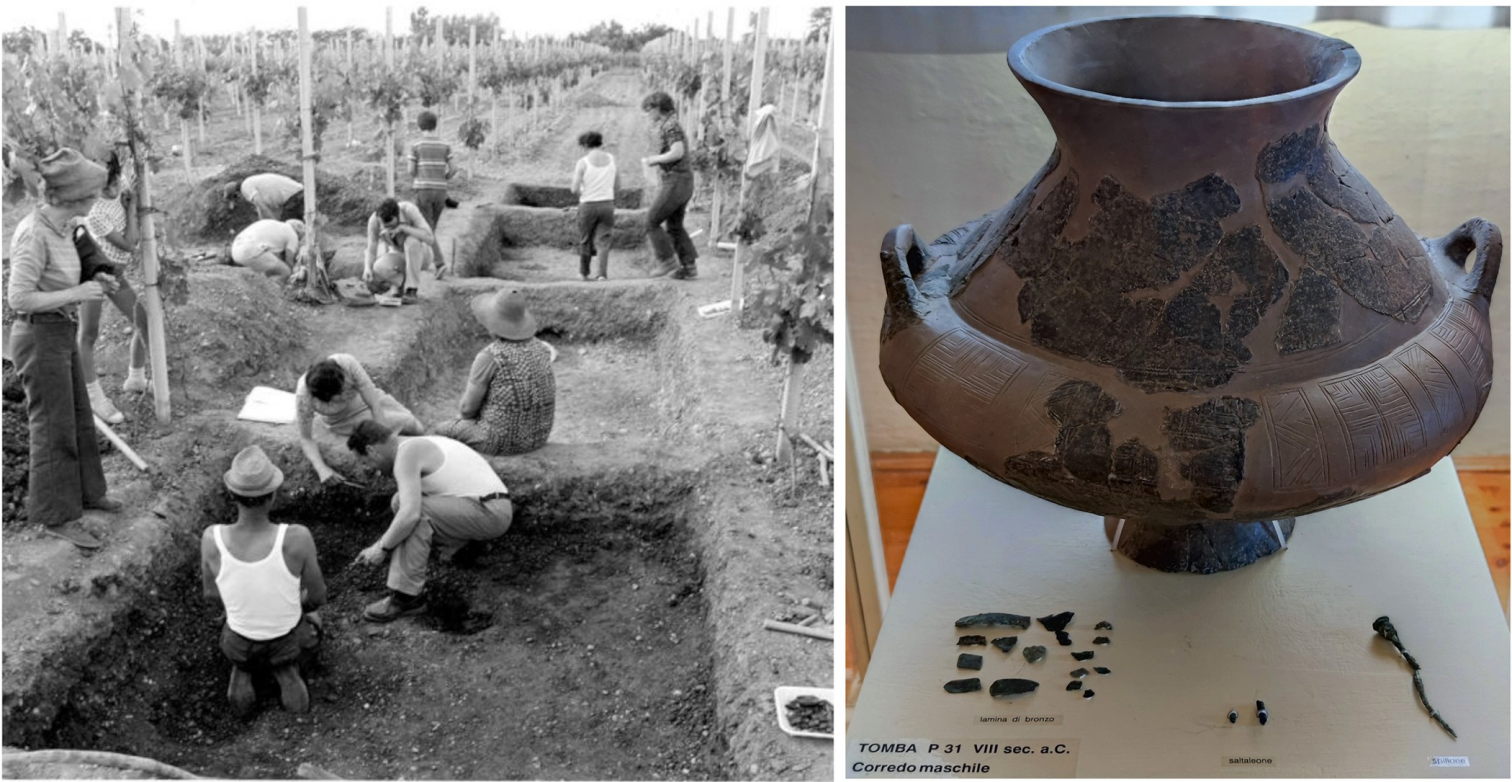
Left: Photo of the excavation of the urnfield of San Valentino in 1973 (Source: [33]; Archivio del Museo Civico di San Vito); right: photo of the biconical urn and the metal objects recovered in Grave P31 as shown in the exhibition of the Museo Civico “Federico De Rocco” in San Vito al Tagliamento (Italy).

The urnfield of San Valentino (lat. 45.91616, long. 12.83729, WGS84) is located 1.5 km west of the present-day town of San Vito al Tagliamento, in the Friuli-Venezia Giulia region, northeastern Italy (Fig 1). In 1972, during preparation for planting a vineyard in the area, a considerable number of metal objects were unearthed. Boosted by these discoveries, archaeological excavations, led by Prof. Paola Càssola Guida from the University of Trieste, began one year later, in 1973, with the aim of identifying the burials and estimating the area of the cemetery [23]. Since the vineyard was already installed, archaeologists were forced to carry out the archaeological excavations in trenches, limited to the spaces between the rows of vines (Fig 2). Fieldwork activities showed that ploughings damaged the graves causing a fragmentation of the urns and dispersion of the cremated remains and the associated grave goods around their original location. Despite that, at least 33 graves and 9 artefact accumulations (referred to as destroyed graves) were identified. However, what remains of the archaeological stratigraphy and the abundant sporadic material recovered suggest that the total number of graves may have been higher [23].

Clearly identified graves were formed by ca. 50 cm-diameter circular pits, without a lining to mark their edges. The urn and, sometimes, the grave goods were placed in the pit, which was then filled with charcoal from the pyre. Ceramic urns were present in two-thirds of the graves (26 out of 33) and contained both the cremated human remains and, in some cases, metal artefacts as grave goods. The metal objects were mainly made of bronze (pins, fibulae, knives, razors, rings, and spiral wires), but iron objects, such as knives, were also present; other characteristic objects include ceramic spindle whorls and glass paste beads. Fragments of metal objects recovered in two graves, probably parts of a cup or strainer, and two bronze carpenter’s tools from the scattered materials, suggest the elevated status of some individuals buried in the urnfield [23, 24]. Finally, two axes and a bronze spear were not recovered in the graves but found out of context. The biconical vessels found in eight graves were not used as urns, but as grave goods, with cremated remains scattered together with metal objects around and under them. Only four graves (D10, D11, G15, N26) lacked ceramic vessels, either as urns or as grave goods, with cremated remains deposited in the grave, probably in a perishable container [23].

The ceramic vessels and bronze objects, which are part of the permanent exhibition in the Museo Civico “Federico de Rocco” in San Vito al Tagliamento (Fig 2), suggest that the individuals buried in San Valentino were not an isolated local community, but had an intense network of relations with other north-Adriatic communities, to the west with the Veneto area and to the east with the S. Lucia group and the inner Carniola region of the Hallstattian area. In particular, the closest contacts are observed with the neighbouring Veneto area, where the biconical vessels, spindle whorls, pins, fibulae, and carpentry tools, find close parallels in some Venetic high-status graves in Este [23, 24] (Fig 1). The lack of weapons, and the presence of axes for clearing forest, small tools related to wood or leatherworking (awls and chisels), and a considerable quantity of ceramic spindle whorls among the grave goods of San Valentino suggest that the community burying their dead in the San Valentino cemetery was distinctly characterized by agriculture and craftsmanship. In this sense, it seems to reflect the large and wealthy community of craftsmen illustrated in the grave Ricovero 236 in Este [23–25]. Similarly to San Valentino, other cemeteries in the Friulan Plain showed contacts with the Venetic area, such as the urnfield of Montereale Valcellina “del Dominu”, at the foot of the mountains north of San Valentino, and the sites of Braida dell’Istituto and Selve at Pozzuolo del Friuli, in the central plain on the other side of the Tagliamento river. The former yielded 21 urn graves in simple pits or in lithic cists and is typologically dated to the 8^th^-7^th^ century BC [26, 27]. The other two date back to the 8^th^-5^th^ century BC [28–30] and consist of 185 and 20 cremation graves respectively, with the cremated remains, the burnt earth, and the grave goods deposited in urns or directly in the grave pits, in some cases covered by a stone slab.

On the occasion of the 50^th^ anniversary of the discovery of the cemetery of San Valentino, this paper provides the first bioarchaeological study, including a dataset of radiocarbon dates on calcined human bones. By integrating the information that strontium isotopes (^87^Sr/^86^Sr) can provide on mobility with the archaeological evidence derived from the study of grave goods, this research aims to understand the nature of the contacts between the society buried at San Valentino and Este and the neighbouring territories, seeking to shed light on one of the most unknown aspects of the EIA in northeastern Italy. In addition, analyses of strontium concentrations ([Sr]) in cremated remains were conducted to try to infer dietary differences, while the cremation conditions (i.e. temperature, fuel, body position, and pyre structure) were investigated by combining FTIR and carbon and oxygen isotope analysis. The C and O isotope and infrared results of San Valentino were compared with the results of the LBA-EIA cemeteries of Blicquy, Grand Bois, Herstal, and Velzeke from Belgium since these are the only published data from this period [14]. Such a comparison has methodological and cultural significance, providing information on variations in the way cremation was practised by different protohistoric contemporary communities belonging to the urnfield phenomenon, even though they were not located in neighbouring territories. The multi-proxy approach applied to this study has never been applied to other cremation cemeteries in the Italian peninsula. In this way, it can open new research possibilities, being potentially extendable to the study of many pre-protohistoric and historic communities that practised cremation.

## Materials and methods

The osteological study of the cremated remains from San Valentino focussed on 21 clearly identified graves, 9 bone clusters not referable to specific graves, the bone deposit identified in 2019, and a bone cluster named “Ex cassa 3” for which the grave number could not be identified (Table 1). The 9 clusters of bones consist of a set of surface-collected bones and 8 assemblages of bone fragments and fragmented objects, recorded as graves in the excavation documentation but inexplicably declassified to simple sets in the first publication [23]. The bone deposit discovered in 2019 and the set of bones designated as "Ex cassa 3" can be linked to individual graves. However, over time, the labels on their containers have become lost, making it impossible to precisely associate them with specific graves within the cemetery. To investigate the mobility dynamics of the individuals buried at San Valentino and to gain new insights into the cremation patterns at the site, 45 calcined bone fragments (S1 Table) were sampled from 19 clearly identified graves, 6 bone clusters in areas D, E, H, and M, the 2019 bone deposit, and the “Ex cassa 3” bone deposit. Nine of these samples (Table 2) were submitted for radiocarbon dating on cremated bone apatite to refine the site chronology. The collection of cremated bones from San Valentino presented many cremation deposits with partially black and grey-coloured fragments. For this study only white (fully calcined) bone fragments were selected because they are resistant to diagenetic alterations due to the increase in crystallinity and therefore suitable for strontium analysis [3]. As a consequence, it was not possible to sample all the excavated graves.

**Table 1:** Results of the osteological analysis on the cremation deposits from San Valentino. For “Ex cassa 3” the grave number could not be identified due to a lack of documentation.

Grave	MNI	Age range	Sex	Total weight (g)	Cranium weight (g)	Long bones weight (g)
2019	1	Adult	ND	1136 g	178 g	356 g
“Ex cassa 3”	1	Adult	ND	61 g	10 g	41 g
Area M, dispersed, corner NE	1	Adult	ND	23 g	11 g	11 g
C1	1	Adult (?)	F	254 g	30 g	163 g
C2	1	Adult	ND	73 g	21 g	45 g
C3	1	Adult	ND	38 g	9 g	27 g
C4	1	Adult	ND	81 g	20 g	51 g
C6	1 (?)	Adult (?)	ND	16 g	2 g	14 g
Area D, dispersed	1	Adult	ND	94 g	3 g	91 g
D8	1	Adult	ND	149 g	8 g	137 g
D 12	1	Adult	ND	48 g	41 g	7 g
D13	1	Adult	ND	22 g	5 g	17 g
Area E, surface survey	1	Adult	ND	19 g	5 g	14 g
Area E, cluster A	1	Adult	ND	22g	8 g	14 g
Area E, cluster B	1	Adult	ND	170 g	32 g	80 g
Area E, cluster E2	1	Non-adult	ND	7 g	3 g	4 g
Area E, cluster E5	1	Adult	ND	301 g	100 g	167 g
E16	1	Adult	ND	101 g	22 g	78 g
E17	1	Adult	ND	56 g	3 g	52 g
G14	1	Adult	F	63 g	5 g	51 g
G15	1	Adult	ND	18 g	13 g	5 g
Area H, W of H18 and H20	1	Adult	ND	30 g	4 g	26 g
Area H, concentration	1	Adult (?)	ND	7 g	2 g	5 g
H18	1	Adult	ND	2 g	/	2 g
H19	1 (?)	Adult	ND	65 g	13 g	52 g
H20	1	Adult	ND	16 g	10 g	5 g
H24	1	Adult	ND	3 g	/	3 g
N25	1	Adult	ND	21 g	9 g	12 g
N27	1	Adult	ND	7 g	1 g	6 g
P28	1	Non-adult	ND	52 g	/	49 g
P30	1	Non-adult	ND	38 g	2 g	36 g
P31	1	Adult	ND	85 g	7 g	70 g

**Table 2:** Radiocarbon dates of fragments of calcined human bone from the cremation deposits from San Valentino.

Grave	Bone fragment	Sample ID	^14^C Lab-code	^14^C Age BP ± σ	Age cal BC (2σ)	Age cal BC (1σ)
2019	Diaphysis	GC0070	RICH-30687	2505 ± 25	776–543 BC	767–568 BC
C6	Diaphysis	GC0096	RICH-30691	2535 ± 25	793–549 BC	787–590 BC
E16	Diaphysis	GC0099	RICH-30695	2812 ± 25	1046–901 BC	1000–928 BC
E17	Diaphysis	GC0082	RICH-30689	2319 ± 25	412–235 BC	403–385 BC
G14	Diaphysis	GC0083	RICH-30690	2726 ± 24	915–815 BC	897–831 BC
H19	Diaphysis	GC0075	RICH-30688	2781 ± 25	1007–837 BC	983–899 BC
N27	Diaphysis	GC0102	RICH-30694	2507 ± 25	776–546 BC	768–569 BC
P30	Cranium	GC0101	RICH-30693	2482 ± 25	771–487 BC	754–545 BC
P31	Diaphysis	GC0098	RICH-30692	2663 ± 24	897–793 BC	829–802 BC

All sampling and analyses were conducted following the ethical standards for the study of archaeological human remains as defined in Italy by Istituto Centrale per l’Archeologia (ICA) and Istituto Centrale per il Catalogo e la Documentazione (ICCD) [34].

Strontium isotope ratios (^87^Sr/^86^Sr) were used to trace human mobility. The ^87^Sr/^86^Sr is correlated to the radioactive decay of ^87^Rb into ^87^Sr and to the initial Rb-Sr ratio of bedrocks. Each geological formation has a specific isotopic signature, related to the age and type of bedrock; older formations (>100 Ma) have a generally higher ^87^Sr/^86^Sr than younger ones [35, 36]. Bioavailable strontium (BASr) is transferred from the geologic system into the soil and plants, and to animals and humans through the trophic chain. Sr isotope ratios measured in calcined bone are used to track the place of geographical origin ([5] and references within). Bone constantly remodels throughout life; this process is called bone turnover and implies that old bone is removed and new bone is formed upon osteocyte signalling which regenerates the skeletal tissues [37–42]. Different skeletal districts exhibit different bone turnover rates, with the rib cortex having the fastest rate [38, 43]. Tooth enamel and the otic capsule of the petrous portion (PP) of the temporal bone are the only tissues that do not remodel after childhood; the PP is not subject to turnover after an individual has reached the age of two years [44–46]. Thanks to these different bone turnover rates in the skeleton, which imply a correlation between Sr intake in human tissues and biological age, it is now possible to use cremated remains to infer human mobility at different phases of life history (e.g. [7]). The PPs of the temporal bones record the Sr intake of the first two years of life, therefore they are used to reconstruct childhood mobility [46, 47]. Adult cranium and long bones, due to their longer turnover, record more recent life stages, corresponding to ca. the last 10–20 years of life history [43, 48].

Nine calcined fragments of cranium, 34 fragments of calcined diaphyses, and two calcined PP were sampled from the osteological remains of the San Valentino cemetery to assess individual and collective mobility through ^87^Sr/^86^Sr (S1 Table). When possible, more than one fragment of cranium and diaphysis were sampled from the same grave to check the possibility of multiple individuals buried in the same grave. Double or multiple cremation burials have been observed in Venetic cemeteries such as Padua and Este [49, 50]. The relevance of using strontium isotopes to investigate the presence of multiple individuals in cremation deposits has been validated by studies conducted in several LBA-EIA cemeteries from Belgium [10, 51]. To identify local and non-local Sr isotopic values measured in the cremated human remains it is necessary to know the local BASr signature. Recently, two ^87^Sr/^86^Sr maps have been released for the Italian Peninsula using different data sources, such as water, foods, sediments, archaeological humans and plants [52, 53]. Since there is a lack of samples from the area of San Vito al Tagliamento, samples of grass, shrubs, and trees were collected from three locations around the site, avoiding agricultural fields, following the methodology suggested by Snoeck et al. [54]. Plants generally contain much more strontium than water and animal foodstuffs, thus contributing larger in the total amount of strontium concentration in the human body [55, 56]. The geology around the site of San Valentino consists of quaternary deposits composed of fluvioglacial and alluvial sediments dating to the Late Pleistocene and the Holocene; sampled locations for plants homogeneously covered these geological units (Fig 1). Strontium concentrations [Sr] were measured in the same bone samples used for ^87^Sr/^86^Sr, with the aim of inferring differences related to diet among the individuals, as recently suggested by Dalle et al. [16, 17] (and references therein).

The cremated samples of cranium and diaphysis that were used for ^87^Sr/^86^Sr and [Sr] were also used for FTIR-ATR and carbon and oxygen isotope analysis. Fourier Transform Infrared Spectroscopy provides useful information into the structural and chemical alterations of bone during cremation using different infrared indices [12, 57–67]; see S1 of Stamataki et al. [14] for more details regarding the FTIR indices). These transformations contribute to our understanding of the cremation conditions during the burning process and provide information on the temperature reached during burning, the ventilation conditions, and/or the presence of garments during cremation [12, 14, 15, 58, 68, 69].

Furthermore, for burned bones, it is possible to measure the carbon and oxygen isotopes of bone apatite carbonates (CO_3_^2-^) and the oxygen isotopes in the phosphate fraction (PO_4_^3−^). After heating and when the bones have been exposed to high temperatures (>600°C) and they are fully calcined, all the water and the organic matter, including the collagen of the human body, are destroyed by the fire and the carbon and oxygen remaining in bone can be found jointly only in the carbonate fraction of bioapatite [12, 70, 71]. Experimental studies have shown that during the burning process, up to 95% of the bioapatite carbon can be replaced by carbon originating from the fuel [68, 72–83]. On the other hand, oxygen exchanges during the burning process are more complicated because there are more sources of oxygen in the burning environment [13, 82, 84]. However, using carbon and oxygen isotope analysis on cremated bones, useful information can be gained regarding the cremation environment and the burning process, which are mostly related to the oxygen availability, the body position on the pyre, the amount of fuel, and the size of the funerary pyre [12–15, 83].

### Osteological analysis

The human remains from San Valentino have always been described as scarce: out ca. thirty bags containing osteological material, only a quarter reached or exceeded ten fragments. This scarcity is due to the disturbed nature of the graves, which were partially destroyed by ploughing implying that only a few bones can be attributed to clearly identified graves. This greatly limited the results of the osteological analysis. The only exception is a concentration of cremated bones, charcoal, and soil recovered in 2019 at the Museo Civico “Federico De Rocco”, inside a bag with a note describing it as a sample from the San Valentino cemetery. This bone assemblage, identified with the number 2019, was also part of the osteological study.

Osteological analysis began with the dry cleaning of the bones, using soft brushes and toothbrushes. Only in a few cases a wet sponge was used to remove the most resistant residues. Then, the bones from each single grave were divided by anatomical district to determine the minimum number of individuals (MNI) [85–87]. A KERN PLE-N precision digital scale was used to calculate their weight, and their degree of representation was obtained both overall and for individual skeletal districts [88]. Due to the small number of fragments, the only two anatomical districts that could be recognised were the cranial and the long bones. Only the 2019 assemblage required initial sieving to separate the human remains from the charcoal and sediments. Subsequently, the bone fragments were dry-cleaned following the same procedure as the other graves.

Estimating the sex and age of a cremated individual is extremely difficult due to the changes caused by heat, which limit the effectiveness of methods traditionally used in bioarchaeology. Given the limited number of remains, sex could only be estimated in very few cases. In those cases, discriminant morphological features [89] and measurements of specific traits [18, 19, 90] were used. It must be specified, however, that fragmentation and deformation can greatly interfere with conventional morphological techniques and osteometric methods. Moreover, the latter are subject to population variability, both temporally and geographically [19, 91]. For this reason, the method of Cavazzuti and colleagues [18] was adopted, as it is based on a partially contemporary population that shared the same geographical area as the San Valentino individuals.

For age at death, given the great fragmentation of the remains and the frequent lack of diagnostic elements, only a distinction between adults and non-adults was possible, based on bone size and morphology. In this regard, account was also taken of the phenomenon of volumetric reduction (between 15% and 30%) that human remains can undergo when exposed to the action of fire [92].

### Radiocarbon dating

Nine samples of fully calcined bone, corresponding to eight fragments of diaphyses and one of cranium that were also used for Sr analyses, were submitted for radiocarbon dating at the Royal Institute for Cultural Heritage (KIK-IRPA) in Brussels, Belgium. Only fully calcined fragments of cremated bone were chosen for ^14^C dating. Black and grey coloured bones were avoided because of the high risk of carbon (C) contamination [74, 79, 80, 83]. The applied protocols for the pre-treatment of the cremated bones, the CO_2_ extraction, and the graphitization are described in Wojcieszak et al. [93] and in Capuzzo et al. [94]. The ^14^C/^12^C ratio in the graphite was measured with the AMS type MICADAS, mini carbon dating system, at the KIK-IRPA (Lab-code: RICH) [95] and converted into a radiocarbon age (expressed in years BP), after correction for isotope fractionation, using the δ^13^C AMS measurement. Calibration of the radiocarbon ages (BP) into calendar years (BC) was performed using the software OxCal 4.4 [96] and the atmospheric calibration curve IntCal20 [97]. To calculate the time spans for the beginning and the abandonment of the cemetery, a chronological model was run in Oxcal 4.4 using simple Boundaries. The duration of the site use was determined using the tool Difference in OxCal 4.4.

### Strontium isotope and elemental analysis

The pre-treatment of 45 samples of calcined bone for strontium isotope analysis (^87^Sr/^86^Sr) was done at the AMGC research unit at the Vrije Universiteit Brussel (VUB), Belgium. The pre-treatment of nine plant samples, strontium extraction from plant and bone samples, and ^87^Sr/^86^Sr measurements were done at the G-Time geochemical facilities at the Université libre de Bruxelles (ULB); elemental concentrations were measured at AMGC (VUB), Belgium.

The selected cremated bone fragments were pre-treated to remove any post-burial contamination. First, all the samples were mechanically cleaned by drilling as described by Stamataki et al. [14]. The petrous portions (PP) of the temporal bones were midmodiolarly cut following the procedure by Veselka et al. [47]. A chemical cleaning was followed adapted from the protocol of Snoeck et al. [3]: 1) six rounds of 10 min of ultrasonication in MilliQ water; 2) one round of 10 min of ultrasonication in 10 mL of 1 M acetic acid; 3) six more rounds of 10 min of ultrasonication in MilliQ water. The samples of calcined diaphyses and cranium were then left to dry at 50°C in an oven for 12 hours and then powdered. The inner cortex of the otic capsule of the PP was sampled as indicated in Veselka et al. [47].

The plant samples were left to dry at room temperature until completely dry. After that, about 500 mg of plant material was placed in a Teflon vial. Samples were acid digested three times with an Anton Paar Multiwave GO Plus microwave digestion system using the Organic B program, which consists of two 10-min phases at 100°C and 180°C, respectively, for a total duration of 40 min including warming steps. The first run was performed with 5 mL HNO_3_ 14M, the second one adding 1 mL HF 23M, and the third one adding 1 mL H_2_O_2_ following the protocol published in Veselka et al. [11]. Once fully dissolved, the solution was transferred to a 7 mL Teflon beaker and left to dry at 100°C on a hotplate. Strontium extraction from powdered calcined bone samples and digested plant samples was performed using columns filled with Sr-specific resin (Eichrom Sr Spec) following the protocol described in Snoeck et al. [3]. ^87^Sr/^86^Sr were measured on a Nu Plasma II MC-ICP Mass Spectrometer from Nu Instruments (Wrexham, UK) at ULB. A standard bracketing method with the recommended value for NIST-SRM-987 of ^87^Sr/^86^Sr = 0.710248 was used to normalise all sample measurements. Procedural blanks were considered negligible (total Sr (V) of max 0.02 versus 7–10 V for analyses, equivalent to ≈ 0.3%). The ^87^Sr/^86^Sr is reported with a 2SD for each sample (absolute error of the individual sample analysis—internal error). Repeated measurements of the NIST-SRM-987 standard returned an average of 0.710247 ± 0.000026 (2SD; n = 86), which is consistent with the mean value of 0.710256 ± 0.000012 (2SD for 88 analyses) obtained by Thermal Ionization Mass Spectrometry (TIMS) [98]. Additionally, measurements of the NIST-SRM-1400 (bone ash) standard yielded ^87^Sr/^86^Sr = 0.713157 ± 0.000022 (2SD; n = 10) which is consistent with the reported value of 0.713117 ± 0.000031 (2SD; n = 345; [99]).

An aliquot of 0.5 mL of the dissolved samples of cremated bone was used to measure the Sr and Ca concentrations. Once diluted again with 0.5 M HNO_3_, a Nu Instrument (Wrexham, UK) ATTOM ES ICP-MS at VUB was used to determine the [Sr] and [Ca] in low and medium resolution respectively using indium (In) as an internal standard and external calibration versus various certified reference materials (NIST-SRM-1400, NIST-SRM-1486). The strontium data were then normalised to 40 wt% Ca to account for the varying loss of organic matter and carbonates during cremation. All [Sr] mentioned in the text and supplementary data refer to Sr concentrations normalised to 40 wt% Ca. Accuracy of the procedure was corroborated by repeated measurements of NIST-SRM-1400 and NIST-SRM-1486. Based on repeated digestion and measurement of these reference materials, the analytical precision of the procedure is estimated to be better than 7% relative standard deviation (1SD, n = 12 for NIST-SRM-1400 and n = 8 for NIST-SRM-1486).

### Carbon and oxygen isotope analysis of bioapatite carbonates

The pretreated archaeological samples of diaphysis and cranium that were used for strontium isotopes and elemental analysis were also used for carbon and oxygen isotope analysis of bioapatite carbonates following the protocol described by Stamataki et al. [14]. All the samples were analysed in duplicates and in total 30 mg of bone powder was used (ca. 15 mg for each measurement). The sealed glass tubes (exetainer® from Labco Limiter) containing the powder were flushed using helium to remove the atmospheric oxygen. Then, 99% phosphoric acid was added to obtain the CO_2_ extraction, which was realised after the reaction of the carbonates present in the bone with the phosphoric acid. The released CO_2_ was analysed with a Nu Perspective IRMS (Isotope Ratio Mass Spectrometer) from Nu Instruments coupled with a Nu GasPrep automatic gas bench at the Vrije Universiteit Brussel (VUB). The results are reported as per mil (‰) deviation from VPDB reference standard [100–102]. Three standards, IA-R022 (δ^13^C = -28.6‰ and δ^18^O = -22.7‰), IAEA-603 (δ^13^C = 2.5‰ and δ^18^O = -2.3‰), and IAEA-CO8 (δ^13^C = -5.8‰ and δ^18^O = -22.7‰), were used to calibrate the isotopic data. Over the course of all analyses, the analytical precision was better than ±0.30‰ and ±0.40‰ (1SD) for both δ^13^C and δ^18^O respectively, based on repeated measurements of in-house cremated bone standard CBA (n = 15; see de Winter et al. [103]).

### Fourier Transform Infrared Spectroscopy in Attenuated Total Reflectance mode (FTIR-ATR)

For FTIR-ATR measurements, 2–3 mg of sieved bone powder (50–25 μm fraction) was used following the protocol of Stamataki et al. [14]. Sieving bone powder has been shown to provide the most reliable and reproducible results [104]. All the samples were analysed in triplicates (ca. 5–6 mg of bone powder) and the infrared indices reported in S1 Table represent the average of three measurements. The infrared analyses were carried out at the VUB-AMGC research unit using a Bruker Vertex 70v FTIR spectrometer under vacuum (spectral range: 4000–400 cm^-1^; number of scans: 32; spectral resolution: 4 cm^-1^; mode: absorbance) and the spectra were analysed using OPUS 7.5 software and all the indices were calculated after the baseline correction (see SI 1 of Stamataki et al. [14] for more details).

Statistical tests were performed using IBM SPSS Statistic version 29.0.1.0 to explore FTIR-ATR and δ^13^C and δ^18^O data and assess the significance among measured skeletal elements of San Valentino. Gaussian normality was checked prior to any statistical comparison using the Kolmogorov-Smirnov test. Since most variables are not normally distributed, the non-parametric Mann-Whitney U test was performed to assess differences among skeletal elements and populations. Statistical significance was set at p≤ 0.05. Principal components analysis (PCA) was conducted using the ‘‘MASS” package [105] in the R programming environment version 4.0.3 [106]. The prcomp function with the argument scale = TRUE, which by default computes principal components based on the covariance matrix, was used. This argument ensures that variables are standardized by subtracting the means and dividing by the standard deviations, representing a form of transformation. Although not explicitly stated, this scaling step is standard practice in PCA to prevent variables with different scales from unduly influencing the analysis. For PCA, all variables were used to compare FTIR-ATR and δ^13^C and δ^18^O data from San Valentino with those from the contemporary Belgian sites of Blicquy, Grand Bois, Herstal, and Velzeke (see Stamataki et al. [14] for more details).

## Results

### Osteological results

Given the scarcity of bone remains, the osteological analysis provided limited results (Table 1). The MNI was found to be 1 for each grave, but caution is needed since this result could be biased by the scarcity of bone material. Regarding sex estimation, only in two cases it was possible to establish a probable female sex (Graves C1 and G14), using exclusively the metric method developed by Cavazzuti and colleagues [18]. In both cases, the anteroposterior and transverse diameters of the axis dens were used. However, it should be noted that, according to Cavazzuti’s method, these measurements have low accuracy. The age at death was adult in almost all graves, except in three cases where non-adults could be identified based on the size and thinness of some cranial bones (Graves P28, P30, and Area E, cluster E2).

The 2019 bone deposit, due to the greater abundance of cremated remains, provided more information. After sieving, no elements were found among the osteological material that might suggest the presence of more than one individual, and no fragments were found whose size might indicate the presence of non-adult remains. Therefore, they could probably be linked to a single adult grave. A trace of greenish circular oxidation was observed on the bone remains, left by a bronze nail recovered in the grave. The age at death of the individual was estimated solely on the basis of bone size, suggesting a tendency towards adulthood. However, no measurable diagnostic features were available to establish sex. A MNI of 1 individual was also estimated for the remains referred to as "Ex cassa 3". The dimensions of the remains are consistent with those of an adult individual.

### Chronology: ^14^C dating

**Fig 3:**
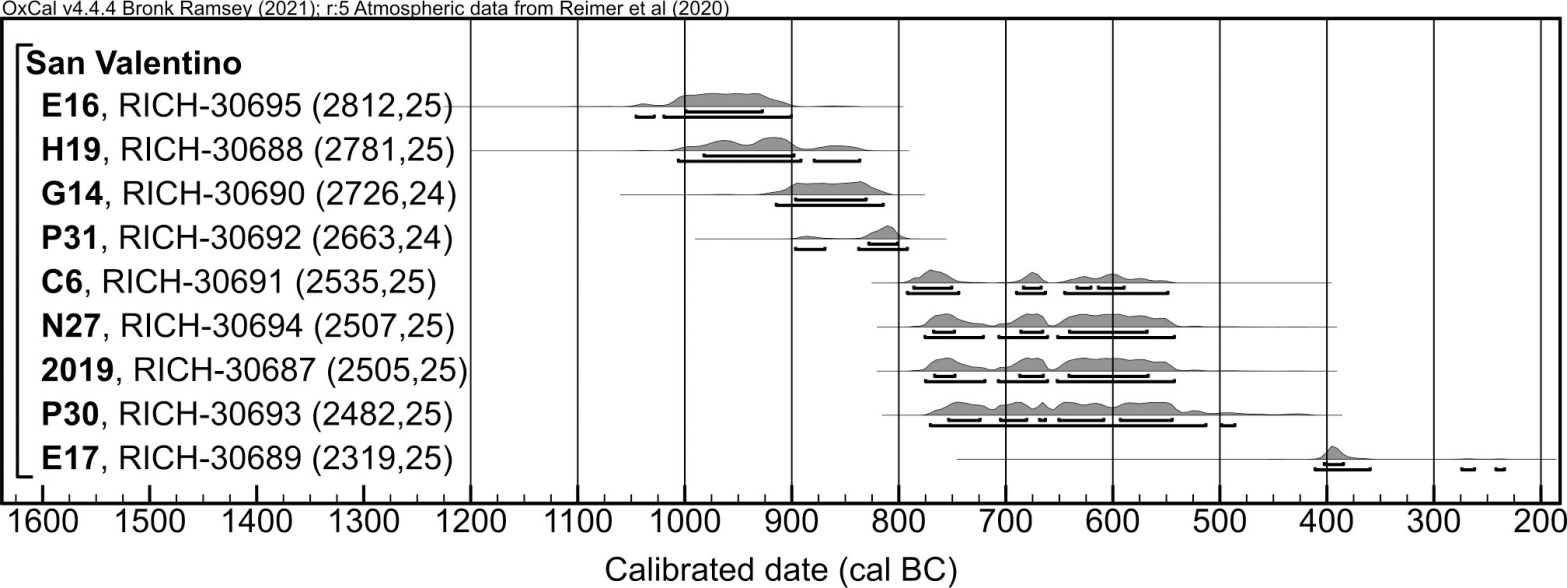
Calibrated radiocarbon dates on fragments of calcined bone from the cemetery of San Valentino.

**Fig 4:**
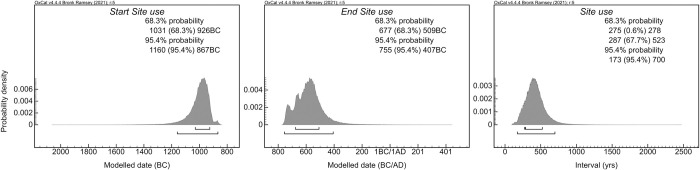
OxCal boundaries estimating, from left to right, the beginning of the cemetery use, its abandonment, and the duration of the site use.

The radiocarbon dates from San Valentino cover a period from the 10^th^ to the beginning of the 4^th^ century BC, with the oldest date (RICH-30695, 2812 ± 25 BP, 1046–901 BC at 2σ probability) associated with grave E16 and the most recent one (RICH-30689, 2319 ± 25 BP, 412–235 BC at 2σ probability) associated with grave E17 (Table 2, Fig 3). The large confidence intervals of four dates (RICH-30687, 30691, 30693, and 30694) are due to the calibration process in the Hallstatt plateau, a plateau in the IntCal20 curve caused by variations in solar activity, which implies large probability distributions covering several centuries for the calibrated dates [97, 107–109]. Given the chronological gap between these ^14^C dates and the most recent date, it could be inferred that the cemetery experienced a phase of abandonment, with grave E17 potentially representing sporadic reoccupation of the funerary area at a later time. The chronological model produced in OxCal, excluding this recent date, places the start of the cemetery use between 1160–867 BC and its abandonment between 755–407 BC, both at 2σ probability, corresponding to the end of the LBA and the EIA. The site would have been in use for approximately 275 to 523 years, with 1σ probability (Fig 4).

### Mobility and landscape use: ^87^Sr/^86^Sr and [Sr] analysis in cremated human bones and biologically available ^87^Sr/^86^Sr baseline

**Fig 5:**
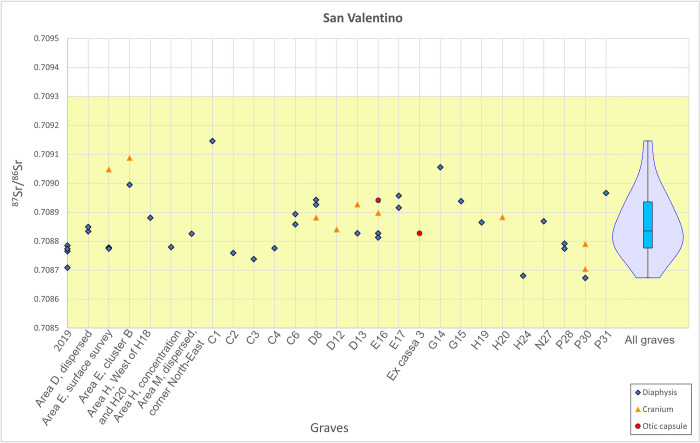
^87^Sr/^86^Sr from fragments of calcined diaphysis, cranium, and otic capsule of the petrous portion of the temporal bone from the individuals buried in the graves of San Valentino and a violin and box plots of average ^87^Sr/^86^Sr from samples of diaphysis in each grave. The BASr range is indicated in yellow.

Samples of cremated bone delivered ⁸⁷Sr/⁸⁶Sr varying between 0.7086 and 0.7091 (S1 Table, Fig 5). Assuming a normal distribution for the ⁸⁷Sr/⁸⁶Sr with the midpoint corresponding to the mean of the distribution and a 2 SD interval, only one sample, from grave C1, could be identified as an outlier. However, using the IQR method (Tukey’s boxplot method), as suggested by Lightfoot and O’Connell [110], for the average ⁸⁷Sr/⁸⁶Sr of the diaphyses in each grave, all samples are within the 1.5xIQR range, indicating that they likely all belong to the same data population (Fig 5). This shows that the strontium isotopic ratios from San Valentino spread over a coherent narrow range. Remarkable consistency (⁸⁷Sr/⁸⁶Sr variations < 0.0003) was observed in the ⁸⁷Sr/⁸⁶Sr from graves that provided multiple samples, supporting the attribution of such cremation deposits to individual graves, as also suggested by the osteological analysis.

The combined use of ⁸⁷Sr/⁸⁶Sr in the inner cortex of the otic capsule of the PP, cranium, and diaphysis was only possible for one individual, allowing us to gain information on the life history, from childhood to adult age, of the individual deposited in grave E16 (Fig 5). The isotopic values span a narrow range (0.7088–0.7089), thus they do not show evidence of large-scale mobility (more than ca. 30 km around the site) of this individual during his/her life.

Local BASr values, measured on plant samples, provided median ⁸⁷Sr/⁸⁶Sr of 0.7082 and 0.7093 for the Late Pleistocene fluvioglacial and alluvial sediments and 0.7089 for Holocene alluvial sediments around the site of San Valentino (Table 3, Fig 1). These isotopic values show the two geological formations are similar regarding their Sr geochemistry. The sample S. Vito 1G yielded a more radiogenic value, which was confirmed after a second measurement, indicating that the plant sample was likely influenced by natural or anthropogenic sources. To estimate the local BASr range, the minimum and the maximum values of the medians were used, which provided the ⁸⁷Sr/⁸⁶Sr interval 0.7082–07093. All ⁸⁷Sr/⁸⁶Sr of human samples, including the two otic capsules of the PP, which are related to the childhood signal, fall within the local BASr range. Therefore, in light of the available isotope data, no non-local individuals were identified in the population buried at San Valentino.

**Table 3:** ^87^Sr/^86^Sr from the plant samples collected in the area around the site of San Valentino.

Sampled location	Latitude	Longitude	^87^Sr/^86^Sr	2SE	Median
S. Vito 1G	45.90004	12.81539	0.711467	0.000008	0.709266
S. Vito 1S	45.90004	12.81539	0.709266	0.000005	
S. Vito 1T	45.90004	12.81539	0.709200	0.000007	
S. Vito 2G	45.90587	12.78580	0.708299	0.000006	0.708200
S. Vito 2S	45.90587	12.78580	0.708200	0.000007	
S. Vito 2T	45.90587	12.78580	0.708075	0.000008	
S. Vito 3G	45.89780	12.89124	0.708861	0.000007	0.708946
S. Vito 3S	45.89780	12.89124	0.708946	0.000007	
S. Vito 3T	45.89780	12.89124	0.709029	0.000007	

**Fig 6:**
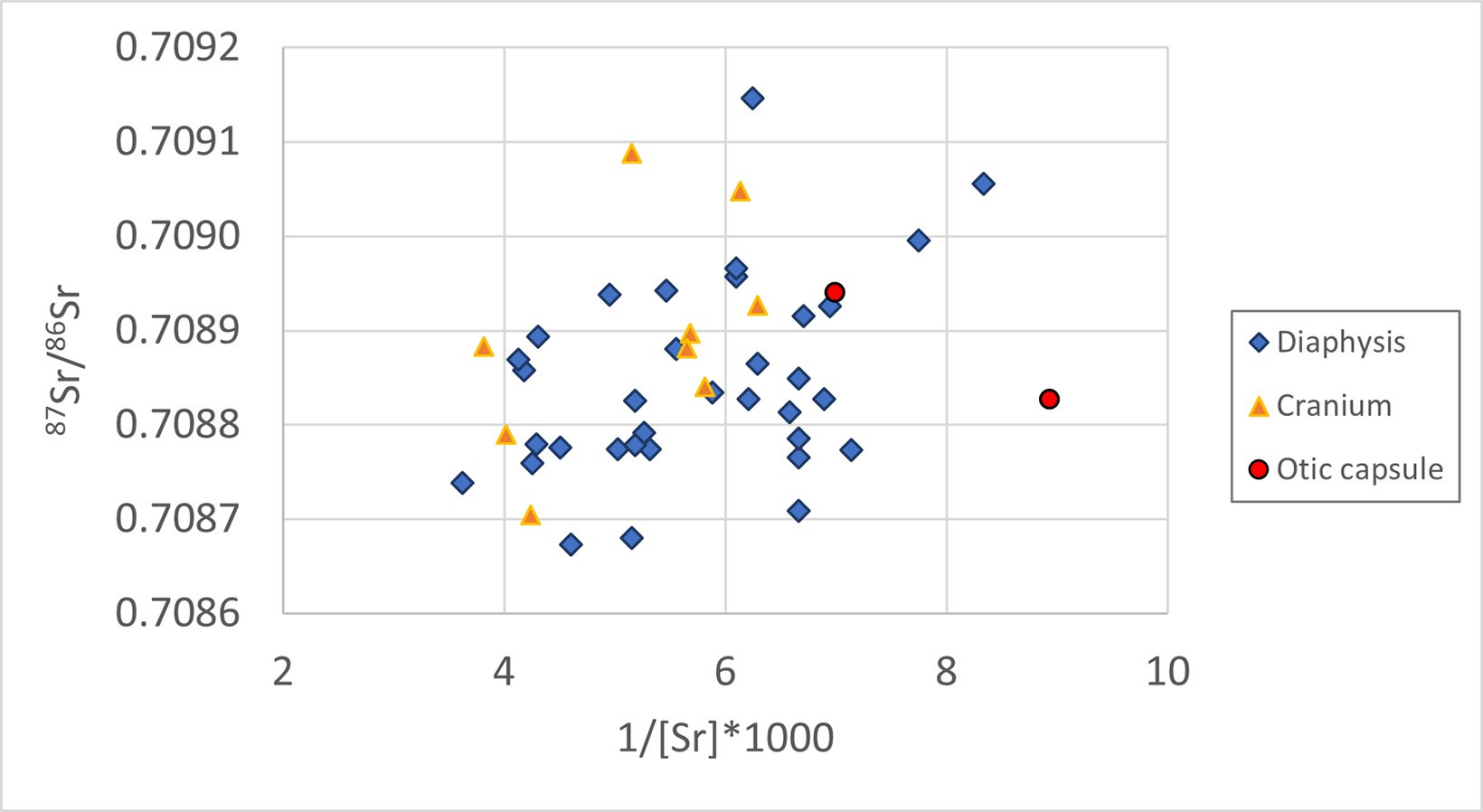
Scatterplot of the Sr results of 1/[Sr]*1000 and ⁸⁷Sr/⁸⁶Sr of fragments of diaphysis, cranium, and otic capsule from San Valentino.

The 45 bone samples produced [Sr] varying between 112 and 276 ppm (S1 Table). No outliers were identified using the IQR method and no variations were observed between diaphyseal and cranial fragments, with samples of diaphysis yielding [Sr] between 120 and 276 ppm, and samples of cranium delivering values between 159 and 262 ppm (Fig 6). The data do not show a linear correspondence between [Sr] and ^87^Sr/^86^Sr (R^2^ = 0.1), suggesting that no variations in subsistence strategies can be identified among the sampled individuals buried in the San Valentino cemetery. Therefore, this indicates that the analysed subset of individuals from San Valentino were a local population that exploited local food resources.

### Funerary practices: Fourier Transform Infrared Spectroscopy and carbon and oxygen isotope analysis

The δ^13^C values of diaphyses (n = 34) vary widely, ranging from -15.1‰ to -24.7‰ and cranial bones (n = 9) from -17.0‰ to -24.0‰ (S1 Table; Table 4). The δ^18^O values of diaphyses also show a high range, between -13.8‰ and -20.9‰, while cranial bones present a narrower range, from -15.5‰ to -18.4‰ (S1 Table; Table 4). However, no statistical differences were observed between the diaphyses and cranial bones (for δ^13^C Mann-Whitney U = 99.5; p = 0.11 and for δ^18^O U = 128.5; p = 0.47).

**Table 4:** Descriptive statistics for C and O isotope and infrared data of diaphyses and cranial bones from San Valentino.

San Valentino Median values	δ^13^C (‰)	Max value	Min value	δ^18^O (‰)	Max value	Min value	IRSF	Max value	Min value	C/C	Max value	Min value
**diaphyses**	-18.6	-13.7	-24.7	-17.0	-13.8	-20.9	4.8	5.2	4.4	1.34	1.53	1.22
**cranium**	-22.1	-17.0	-24.0	-16.9	-17.0	-18.4	5.3	6.3	4.8	1.33	6.35	1.20

**Fig 7:**
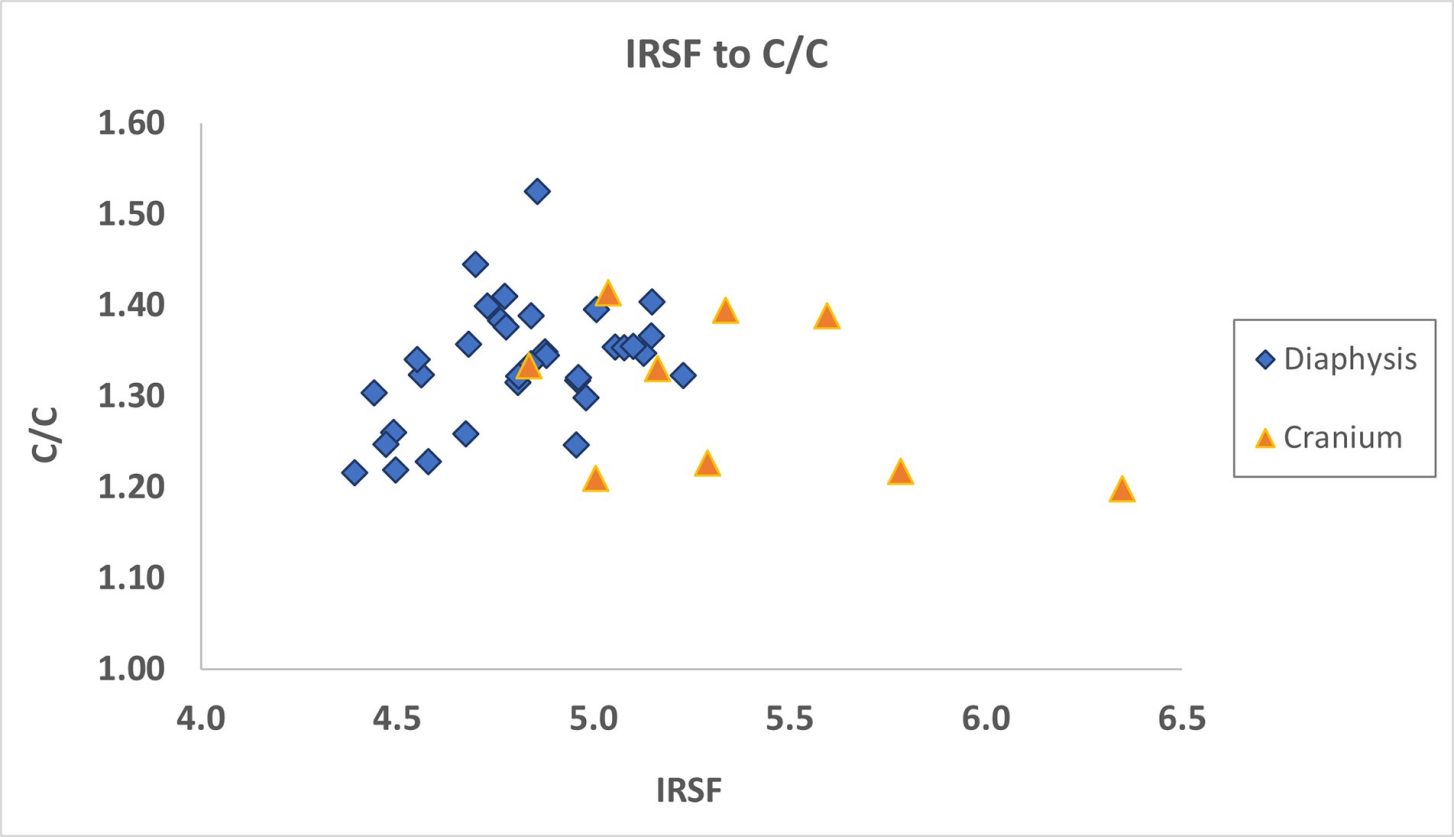
Scatterplot of IRSF to C/C values of diaphyses and cranial bones from San Valentino.

Regarding the infrared values, diaphyses present a narrower range in infrared splitting factor (IRSF) values (from 4.4 to 5.2) compared to cranial bones (from 4.8 to 6.3), with the cranial bones presenting the highest IRSF values (S1 Table; Table 4, Fig 7). Mann-Whitney U statistical test between diaphyses and cranial bones indicated that there is a statistically significant difference in IRSF values (U = 268.0; p < 0.01), but no statistical differences were observed in carbonyl-to-carbonate ratios (C/C) between the two skeletal elements (U = 120.0; p = 0.33) (Fig 7).

In addition, the extremely low values of the amide-to-phosphate ratios (Am/P <0.005) in both diaphyses and cranial bones, in combination with the high crystallinity (IRSF values from 4.4 to 6.3), suggest the absence of any organic matter and/or adsorbed water in the bone structure. This could indicate that the bone fragments selected for this study were likely exposed to high-temperature burning, resulting in fully calcined bones.

**Fig 8:**
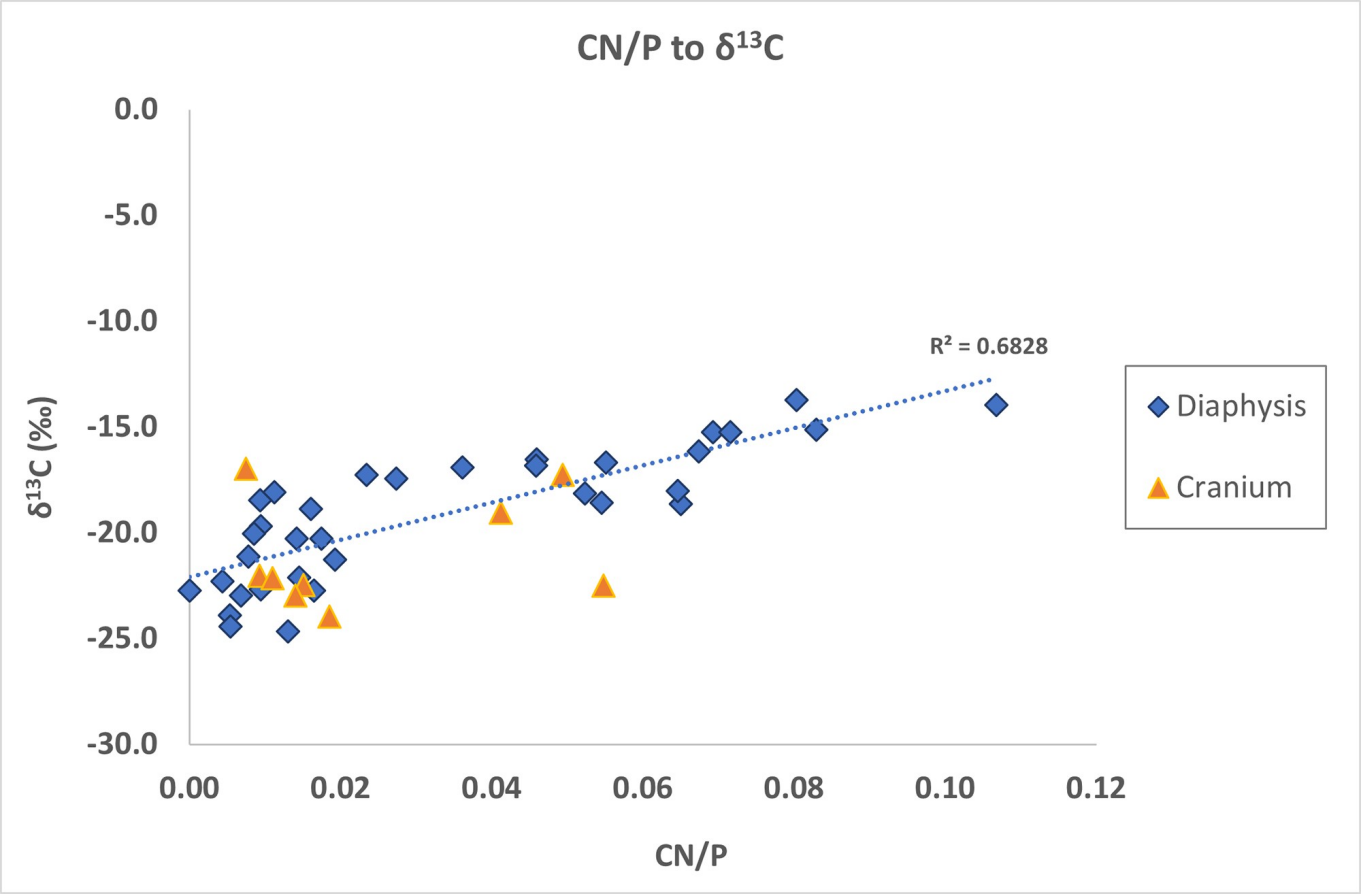
Scatterplot of CN/P ratios to δ^13^C values of diaphyses and cranial bones from San Valentino.

It was also observed, as shown by Snoeck et al. [13], that all the samples containing cyanamide (H_2_CN_2_) (CN/P > 0.02; see Salesse et al. [15] for more details) generally have higher δ^13^C values but no significant difference in δ^18^O values (S1 Table). There is a linear correlation between CN/P and δ^13^C (R^2^ = 0.68) in diaphyseal samples (Fig 8). Specifically, 16 out of 34 diaphyses had CN/P > 0.02, representing 47% of the diaphyses. The presence of cyanamide could be related to a limited exchange and incorporation of fuel carbon into the bone apatite structure. As a result, the cyanamide-to-phosphate ratio (CN/P) has been linked to low oxygen availability during burning [12, 68] and to the co-existence of ammonia during combustion, which could be linked to the potential presence of garments worn by the deceased during cremation [15] or a large pyre. Finally, no statistical differences were observed in CN/P ratios between the diaphyses and cranial bones (Mann-Whitney U = 131.5; p = 0.52).

## Discussion

The combination of state-of-the-art methods in biogeochemistry applied to the analysis of cremated remains has allowed to shed light on the chronology, human mobility, and funerary practices of a preserved sample of individuals buried in the urnfield cemetery of San Valentino, in northeastern Italy.

The new high-resolution radiocarbon dates have refined and confirmed the site chronology, previously based only on the typo-chronological analysis of grave goods, which cover a time range between the end of the LBA (10^th^ century BC) and the EIA (9^th^-7^th^ century BC) [24]. The modelling of the ^14^C dates showed that the occupation of the burial area started in the 10^th^ century BC and ended in the 7^th^-6^th^ century BC, with a sporadic reoccupation at the turn of the 4^th^ century BC. The typological dates of the grave goods recovered in the ^14^C-dated graves are, in almost all cases, in good agreement with the radiocarbon results. Grave H19 yielded fragments of a biconical urn (fig. 8, B-D in [23]), which finds parallels with types datable to the Este I and IIA-B phases (ca. 950–830 BC). For the chronological framework based on the Este cemeteries, see the following references [25, 111, 112]. In Grave G14, a vase-headed pin was recovered fig. 6, tomba G14, 2 in [23]), which shows typological similarities with pin n. 1996 in Carancini [113], which is referable to type VI, variety B, of Peroni and Vanzetti (p. 55 in [114]), assigned to the Bologna IIA phase (ca. 880–830 BC). Grave P31 yielded a vase-headed pin (fig. 14, tomba P31, 2 in [23]), referable to type IV, variety B, of Peroni and Vanzetti (p. 55 in [114]), also assigned to the Bologna IIA phase, and a biconical urn (Fig 2) (fig. 10, C in [23]), which finds parallels with types of the Este I and IIA-B phases. The biconical urn in Grave N27 (fig. 13 in [23]; fig. 5,1 in [114]) was typologically dated to the same phase. This grave also yielded two fragments of a bronze razor assigned to the end of the 8^th^ century BC (fig. 11, tomba N27, 3 in [23]; fig. 5,4 in [115]). Grave C6 provided two fibulae with foliate arch (fig. 5, tomba C6, 2–3 in [23]), typologically dated between the 8^th^ and the third quarter of the 7^th^ centuries BC (phases Este II-IIIB1). Grave P30 provided a Vadena-type pin (fig. 14, tomba P30, 3 in [23]), referable to type II, variety B, in Peroni and Vanzetti (pp. 56–57, tav. 5 in [114]), assigned to the Este IIC-IIIA phase (ca. 830–700 BC). Grave 2019 contained a cranial fragment with traces of oxidation due to contact with an iron object, a bronze nail, and many beads in yellowish glass paste, which are attested in Venetic burials from the end of the 7^th^ century BC, such as the rich grave Benvenuti 126 from Este (tav. 176, 6 in [111]). The ^14^C date of Grave E16 is slightly older than the typological date derived from the analysis of grave goods, which include a fibula with a lowered bow decorated with incisions (fig. 7, tomba E16, 2 in [23]), whose use is documented from the Este IIA phase (ca. 880–820 BC) [114]. The ^14^C-dated fragment of calcined bone could either be affected by an old-wood effect or correspond to an intrusion from another grave, as the date fits within the time span of use of the cemetery.

Unexpectedly, a later occupation of the burial area at the turn of the 4^th^ century BC was documented by the radiocarbon date on a calcined bone from Grave E17, whose grave goods, however, suggest a much older chronology. These include a Capodaglio-type pin (fig. 7, tomba E17, 2 in [23]), whose use is documented from the Este IIC phase (ca. 830–730 BC) [114], and two fragments of iron knives datable to the EIA (fig. 7, tomba E17, 3–4 in [23]). Until now, the area around San Vito has not yielded materials subsequent to the EIA and prior to Romanization. Overall, the transition to the Late Iron Age (LIA) seems to coincide with the beginning of a period of generalised crisis for the territory of the Friuli plain. West of the Tagliamento river, the passage from the 5^th^ to the 4^th^ centuries BC corresponds to the final moment for the protohistoric settlements preceding Roman colonisation. To that period is dated the abandonment of the enclosed settlement of Gradisca di Spilimbergo, north of San Vito, while in the southern area around the protohistoric Venetic settlement and later Roman city of Concordia there is no archaeological evidence between the 5^th^ and 2^nd^ centuries BC. Such lack is not well understood today, given the evident continuity between the Venetian and Roman phases in the nearby eastern Veneto settlements of Oderzo and Altino, where the LIA phases are well documented [116–121].

We cannot exclude that, given the heavy agricultural activities that took place in the area of the cemetery, with the removal of soil, ploughing, and subsequent planting of vineyards, a further archaeological phase from the Late Iron Age was destroyed. In today’s western Friuli, in the northern mountain area known as Carnia, cremation graves datable between the late 8^th^ and early 4^th^ century BC were recognised in the necropolis of Misincinis di Paularo. At the same site, a further occupation belonging to the La Téne culture (Late Iron Age) was recognised in the disturbed surface layer above the oldest tombs [24]. Grave E17 was disturbed at the time of excavation, and an attribution of the dated bone fragment to probable contamination from upper layers that no longer exist cannot be excluded.

The analysis of strontium isotope ratios in cremated human remains has not distinguished non-local individuals in the studied graves. Currently, no secure archaeological evidence of the settlement related to the San Valentino cemetery is available. This information is relevant in attempting to determine the possible areas from which the harvested crops contributed through the trophic chain to the ⁸⁷Sr/⁸⁶Sr of the individuals buried in the urnfield. During the Bronze and Iron Ages, these fields were likely located in the surroundings of the settlements. In that period, the settlement system in use in the Friuli Venezia Giulia area, between the Livenza river to the west and the Karst plateau to the east, consisted of enclosed settlements or hillforts, the so-called *Castellieri*, which were located in the alluvial Upper plain of Friuli, rarely on hilltops, characterised by earth ramparts and surrounded by a moat [122, 123]. The only settlement around San Valentino, whose chronology partially matches that of the urnfield, is the *Cjastelar* (literally “hillfort” in the local language) of San Giovanni di Casarsa [33, 124], which is located 3.3 km north of the urnfield. The site was deeply affected by agricultural activities that flattened the hillfort and hindered the possibility of carrying out archaeological stratigraphic excavations. Surface surveys yielded materials spanning the LBA to the EIA (12^th^-9^th^ century BC). This settlement sits on the same geological formation identified for the San Valentino cemetery, formed by fluvioglacial and alluvial sediments dating to the Late Pleistocene, whose BASr baseline ranges from 0.7082 to 0.7093.

The Friulian Plain and the Po valley are constituted by Pleistocene-Pliocene and Holocene deposits formed by glacial and alluvial sediments, whose ⁸⁷Sr/⁸⁶Sr reflect the admixture of the ⁸⁷Sr/⁸⁶Sr that characterise the different geological units affected by the erosive activity of rivers in the uplands [6]. Recently, a Sr isoscape has been produced for the Italian Peninsula using a large variety of bioavailable and non-bioavailable samples, such as plants, water, biominerals (i.e., bones, teeth, and bio calcareous shells), food, soil, and rocks [53], which has improved the resolution of the previous Sr map in Emery et al. [52]. This BASr isoscape identifies lower ⁸⁷Sr/⁸⁶Sr in the eastern Friulian plain and higher values in the western part comprising the Veneto Region; the estimated ⁸⁷Sr/⁸⁶Sr in the area around San Vito are in agreement with those presented in this paper. The map is a valid starting point to understand Sr variability in the Italian Peninsula, although the amount of plant samples, which reflect the primary source of bioavailable strontium in humans, is still reduced, while the majority of proxies used to develop the Sr isoscape are food, water, and biominerals, which all have pros and cons for tracking human past mobility [125]. The area around Este, in the Veneto region, is characterised in the Italian Sr isoscape by slightly higher ⁸⁷Sr/⁸⁶Sr compared to those measured around the San Valentino site. Additionally, ca. 30 km southwest of Este, in the municipality of Legnago (Fig 1), the Scalvinetto site is located, which is one of the largest bi-ritual cemeteries of the Terramare culture north of the Po river, whose occupation spans the Middle and Late Bronze Age (ca. 1450–1000 BC) [126–128]. ⁸⁷Sr/⁸⁶Sr from the cremated individuals buried at Scalvinetto have been recently measured, yielding values ranging from 0.7092 to 0.7103 [6]. The site is placed on Holocene and Late-Pleistocene alluvial sediments, and the local BASr baseline has provided values in the range 0.7096–0.7101, which is higher than the BASr baseline measured in the area around San Valentino. All these results support that we can exclude the provenience of the analysed individuals buried at San Valentino from the area of Este; the similarity in pottery and metallic forms with those recovered in the graves of Este would therefore be the consequence of a transfer of ideas and models rather than the result of episodes of people movements.

The variability in cremation settings within the cemetery of San Valentino is examined to investigate potential differences in the way cremation was performed on a local scale. It is worth mentioning that the differences in burning conditions based on biological sex could not be examined at San Valentino because of the high number of indeterminate-sex individuals, while only two individuals were assessed as female and none as male. Furthermore, age-related differences in cremation settings could not be investigated because of the low number of identified non-adults (only three). Interestingly, there were no observable intra-site differences in burning conditions between the earlier and the later chronological phases despite the long-term use of the cemetery.

Intra-site variations were detected between diaphyses and cranial bones in IRSF values but not in C/C ratios (S1 Table; Table 4, Fig 7). These two indices have been shown to be temperature dependent [12, 58, 67, 69, 129]. In the case of San Valentino, it seems that there are no temperature-related differences between diaphyses and cranial bones since differences were only observed in IRSF values and not in C/C ratios. The equal burning of the different body parts could be related to the size of the funerary pyre and/or the amount of wood used during cremation. A large funerary pyre where the whole body would be evenly impacted by the fire could explain the high IRSF values on cranial bones. Another explanation could be related to the management of the funerary pyre and the person(s) who were performing the burning process. This person (i.e. the cremator or cremation operator) could have been a ritual specialist, a skilled worker (e.g. a smith or a potter) [130–133], or even a family member [134], who made sure that the whole body was exposed to the fire and that even the cranium, which was at the edges of the funerary pyre, was fully burned, resulting in higher IRSF values for cranial bones.

The presence of cyanamide (Fig 8: CN/P to δ^13^C) has been linked to low oxygen availability during combustion, to the position of the body on the funerary pyre, and/or the presence of ammonia during cremation [12, 15, 67, 68]. Considering that 47% of all diaphyses present cyanamide (16 out of 34 diaphyses), it can be assumed that the presence of cyanamide in San Valentino diaphyses is probably linked to the body’s position on the funerary pyre or the size of the pyre, which could lead to lower oxygen availability for some of the diaphyses during combustion if the body of the individual was in the middle of the pyre instead of on top, based on the study of Salesse et al. [15]. Another alternative could be related to the person(s) who was managing the funerary pyre during cremation, which could have affected the way diaphyses were burned. However, more research is necessary to better understand the position of the body on the pyre and/or the pyre management.

The lack of difference in δ^13^C (‰) and δ^18^O (‰) values between diaphyses and cranial bones indicates, like the infrared data, that the whole body was probably burned in a similar way. Furthermore, differences were not observed among the cremated individuals, demonstrating that there was probably no variation in the type and/or amount of fuel used for the funerary pyres in San Valentino nor any difference in oxidation conditions during combustion.

**Fig 9:**
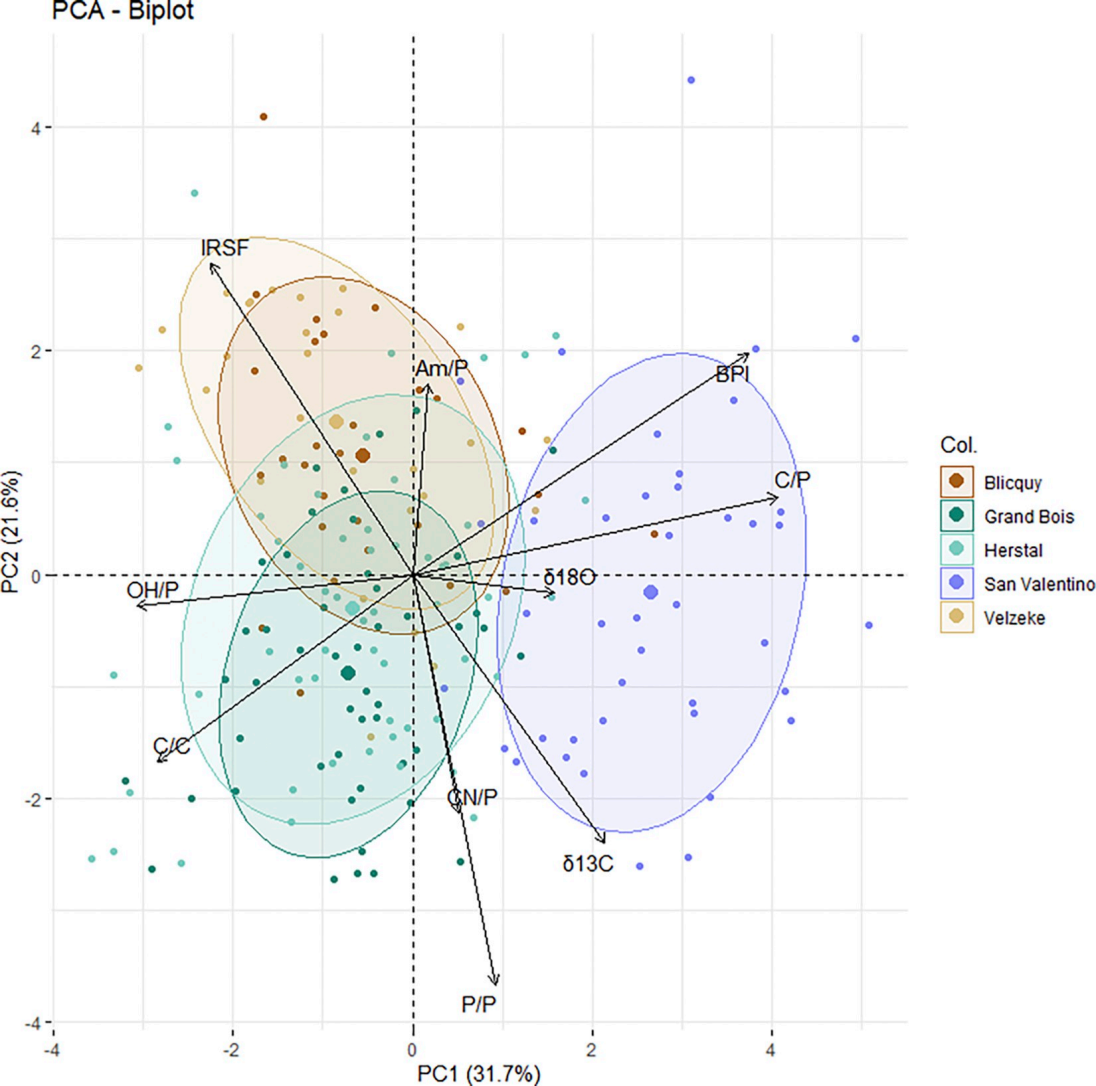
Principal components analysis (PCA) results among the Belgian sites (Blicquy, Grand Bois, Herstal, and Velzeke [14]) and San Valentino using a set of infrared variables (IRSF, C/C, BPI, C/P, CN/P, P/P, OH/P, and Am/P) and isotopic data (δ^13^C and δ^18^O).

Relevant information on methodological and cultural aspects related to how cremation was practised by different communities inhabiting Europe at the end of the Bronze Age and during the EIA can be gained by the comparison of the C and O isotope and infrared results of San Valentino with the results of the LBA-EIA cemeteries of Blicquy, Grand Bois, Herstal, and Velzeke from Belgium [14] (Fig 9; Table 5). Stamataki et al. [14] demonstrated that it is possible to assess the inter- and intra-site variability in cremation settings using a multi-proxy analysis which combines FTIR-ATR data with C and O isotope analysis. Thanks to this approach the authors [14] managed to identify clear differences in the cremation settings between funerary sites located in two distinct cultural area during LBA-EIA in Belgium: the Scheldt Basin (sites of Blicquy and Velzeke) and the Meuse Basin (sites of Grand Bois and Herstal) which were not possible to detect by osteological analysis alone. In this way, it was demonstrated that the burning process was not homogeneous in the Belgian region, but it confirmed the cultural influences observed in ceramic and bronze artefacts in the two basins.

**Table 5:** Median values and interquartile ranges (IQR = Q3-Q1) of δ^13^C, δ^18^O, IRSF, and C/C per skeletal element and site.

**Median δ** ^**13**^ **C values per skeletal element (Q1 to Q3) (‰)**					
**Skeletal element**	**San Valentino**	**Velzeke** [14]	**Blicquy** [14]	**Grand Bois** [14]	**Herstal** [14]
**Long bones**	-18.6 (-22.1 to -16.9)	-21.8 (-25.1 to -20.9)	-23.4 (-24.2 to -20.6)	-21.4 (-23.5 to -20.8)	-22.5 (-23.7 to -21.1)
**Cranium**	-22.1 (-22.5 to -19.0)	-25.4 (-25.7 to -23.7)	-24.0 (-25.1 to -22.0)	-22.4 (-24.0 to -20.9)	-22.3 (-23.8 to -21.9)
**Median δ** ^**18**^ **O values per skeletal element (Q1 to Q3) (‰)**					
**Long bones**	-17.0 (-17.6 to -15.6)	-18.2 (-19.2 to -17.8)	-19.0 (-19.5 to -18.0)	-18.5 (-19.0 to -17.9)	-18.2 (-18.8 to -16.7)
**Cranium**	-16.9 (-17.9 to -16.6)	-18.3 (-19.3 to -17.8)	-17.5 (-18.5 to -16.8)	-19.2 (-20.2 to -17.9)	-18.1 (-20.4 to -17.2)
**Median IRSF values per skeletal element (Q1 to Q3)**					
**Long bones**	4.8 (4.7 to 5.0)	5.8 (5.5 to 6.2)	5.94 (5.7 to 6.1)	5.28 (5.1 to 5.5)	5.18 (5.0 to 5.3)
**Cranium**	5.3 (5.0 to 5.6)	6.3 (6.0 to 6.5)	5.79 (5.5 to 6.2)	5.19 (5.0 to 5.4)	5.48 (5.2 to 5.7)
**Median C/C values per skeletal element (Q1 to Q3)**					
**Long bones**	1.34 (1.30 to 1.38)	1.42 (1.35 to 1.52)	1.46 (1.32 to 1.66)	1.51 (1.31 to 1.95)	1.51 (1.22 to 2.16)
**Cranium**	1.33 (1.22 to 1.39)	1.40 (1.31 to 1.45)	1.44 (1.32 to 1.55)	1.46 (1.31 to 1.59)	1.48 (1.25 to 1.82)

The investigation of chemical and structural changes occurred during cremation, using FTIR-ATR data and C and O isotope data applied to cremated remains, has never been applied to any other Italian archaeological site except San Valentino. For this reason, the only possible comparison is with the coeval Belgian sites of Blicquy, Grand Bois, Herstal, and Velzeke, which are currently the only LBA-EIA sites on which these kinds of data are available. This comparison has a methodological relevance since Belgian data can be used as “baseline” to interpret the data from San Valentino and also a cultural relevance since it provides important information regarding the differences in cremation conditions (e.g. temperature, pyre structure, location of the pyre in the environment) among contemporary sites belonging to the urnfield phenomenon which are, however, located in different geographical and non-adjoining regions. For this reason, PCA was conducted using all FTIR-ATR (IRSF, C/C, BPI, C/P, CN/P, P/P, OH/P, and Am/P) and stable isotope (δ^13^C and δ^18^O) variables and using the prcomp () function in R. PCA is sensitive to the relative scaling of the original variables, so before PCA analysis, all variables were also transformed using the function “caret”. In general, the multivariate statistical results suggest that both infrared (mostly IRSF, C/C, OH/P, P/P, C/P, and BPI) and isotope data from Belgium and Italy (San Valentino) present differences. More specifically, three clear clusters are formed (Fig 9). The cluster of San Valentino is differentiated from the two Belgian clusters (see Stamataki et al. [14] for more details about the differences in Belgian cremation conditions during the LBA-EIA) indicating possible differences in cremation conditions among the four Belgian sites and San Valentino. This means that the way cremation was practised at San Valentino does not correspond to the way it was practised by the contemporary protohistoric communities inhabiting the Belgian Scheldt and Meuse basins, but it presents specific characteristics.

**Fig 10:**
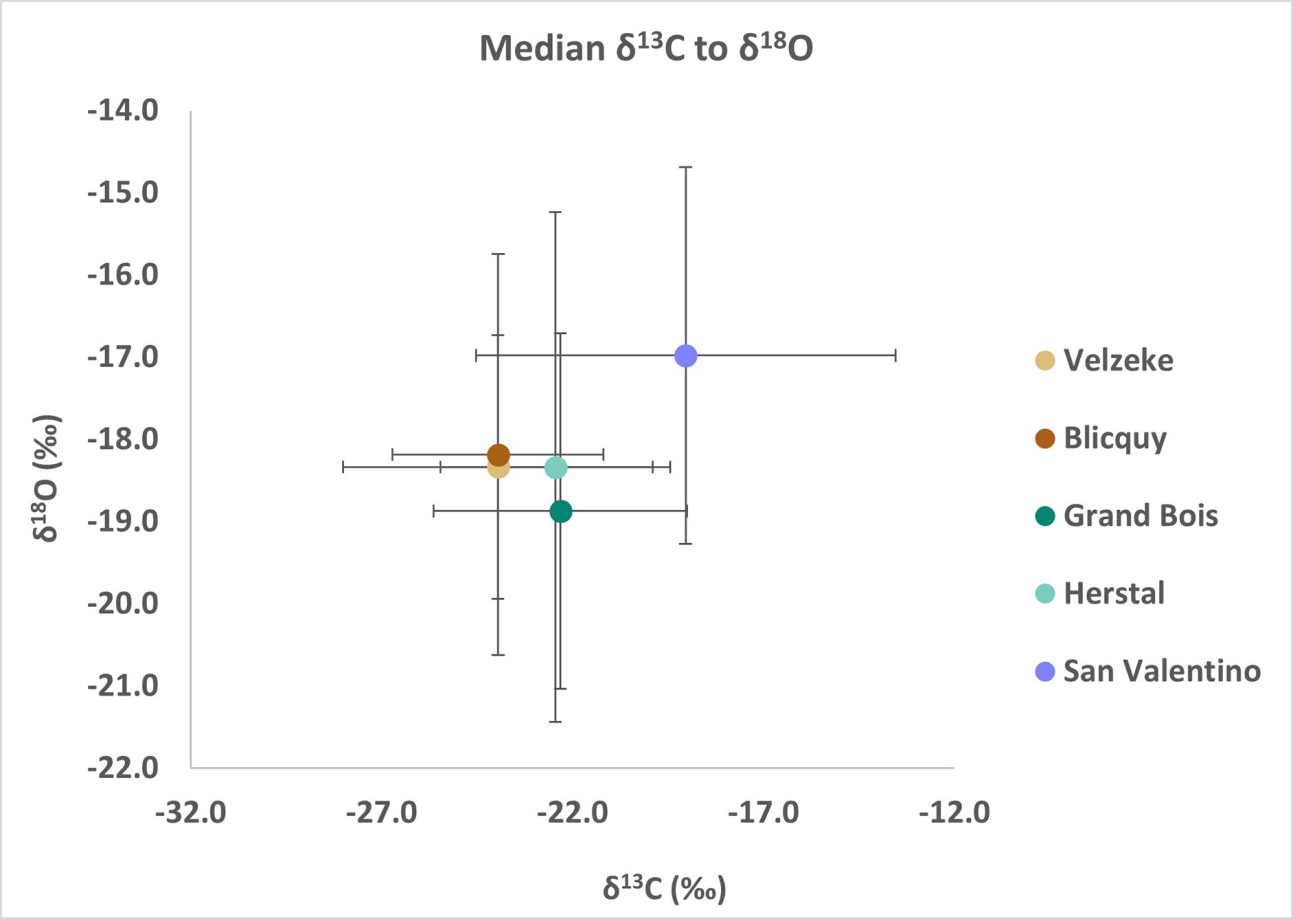
Median δ^13^C and δ^18^O values of diaphyses and cranial bones from Italy (San Valentino) and Belgium (Velzeke, Blicquy, Grand Bois, and Herstal; Stamataki et al. [14]) from the LBA-EIA. The error bars indicate the 1^st^ and the 3^rd^ quartiles.

Based on the carbon and oxygen isotope analysis, median δ^13^C and δ^18^O values are generally higher (ca. 4‰ for δ^13^C and 1‰ for δ^18^O) in San Valentino compared to the Belgian sites (Fig 10; Table 5), and this difference is statistically significant (for carbon: Mann-Whitney U = 1818.5; p < 0.01; for oxygen U = 1854.5; p < 0.01). These higher δ^13^C values, as well as the greater variability in the range of δ^13^C values observed in San Valentino, could be related to the use of different types of wood [135, 136], and/or different amounts of fuel used to build the funerary pyre. The differences in δ^18^O values could be attributed to many factors such as the position of the body on the pyre (e.g. on the top vs in the middle vs at the bottom of the pyre), the size, the structure (e.g. good structure with ventilation vs poor structure with low oxygen availability), the location of the pyre in the environment (e.g. on a hill vs in a valley), or even the season and the amount of wind during cremation, and the atmospheric δ^18^O values of different countries at different latitudes. The location of San Valentino close to the sea (ca. 30km in a straight line) could be related to better ventilation conditions during the burning process and might explain the higher δ^18^O values. More research is, however, needed to understand how the factors above affect the δ^13^C and δ^18^O values during combustion.

**Fig 11:**
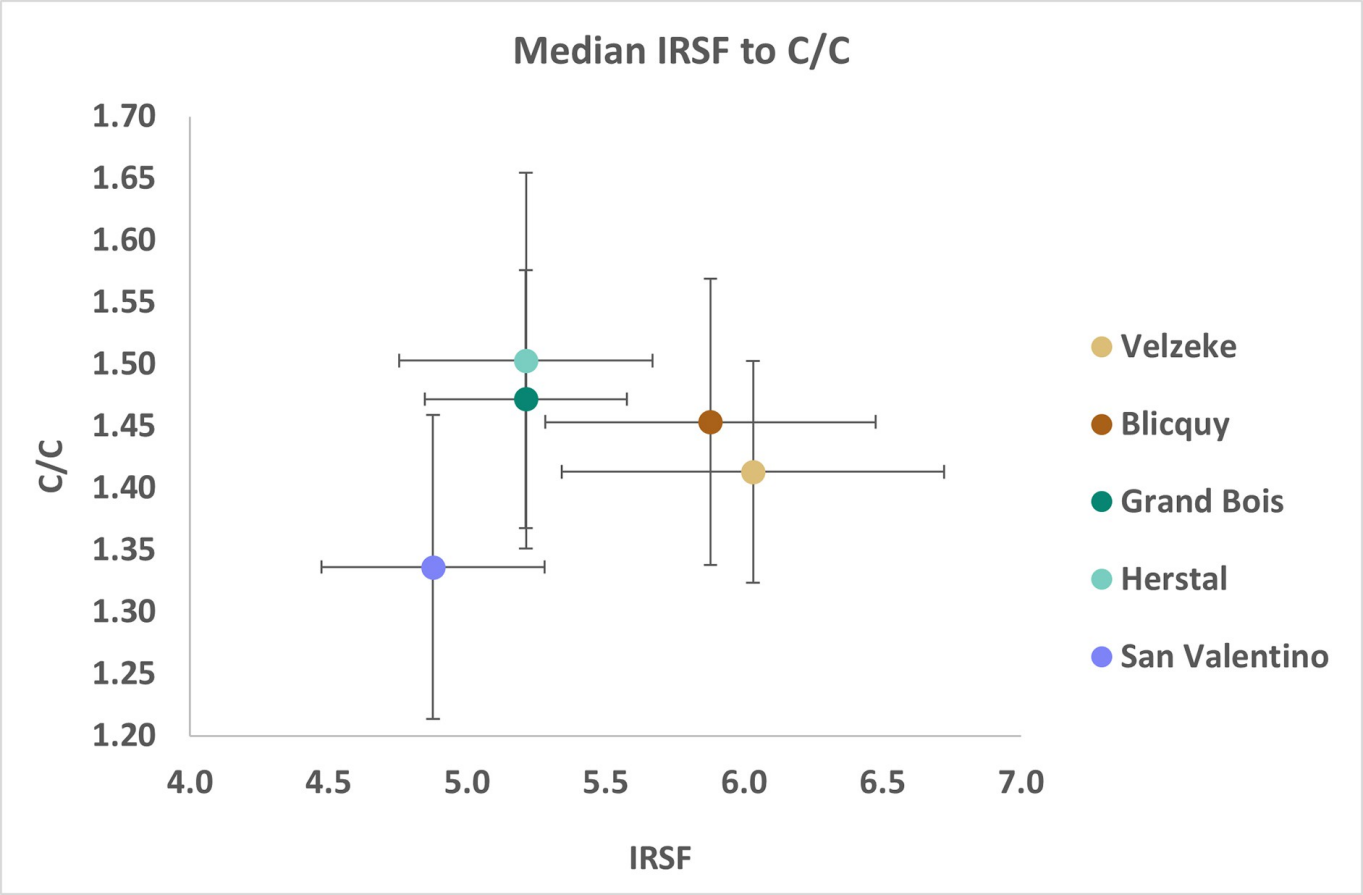
Median C/C to IRSF values of cremated bones from Italy (San Valentino) and Belgium (Velzeke, Blicquy, Grand Bois, and Herstal; Stamataki et al. [14]) from the LBA-EIA. The error bars indicate the 1^st^ and the 3^rd^ quartiles.

Regarding the infrared results, the cremated bones from San Valentino present lower median IRSF values and C/C ratios compared to the Belgian sites (Fig 11). The Mann-Whitney U statistical test between San Valentino and the Belgian sites of Velzeke, Blicquy, Grand Bois, and Herstal indicated that there are statistically significant differences for both IRSF (U = 1508.0; p < 0.01) and C/C ratios (U = 1017.0; p < 0.01). These differences are likely related to the temperatures reached during cremation in the different sites, suggesting that the burning temperature was generally lower at San Valentino than in the Belgian sites despite the good ventilation conditions indicated by the high δ^18^O values. This conclusion is supported by the presence of grey and partially black bones in the osteological collection from San Valentino. The use of different kinds and/or amounts of wood for the funerary pyre between San Valentino and the Belgian sites, as already suggested by the variability observed in the δ^13^C values, or the better management of the pyre during the burning process, could explain the higher temperatures of combustion in the Belgian sites. The study of more Italian sites from the same chronological period is, however, important for understanding the way cremation was performed in Italy during the LBA and the EIA, a period in which cremation was the dominant funerary rite.

## Conclusion

This study demonstrates how a multi-proxy approach, which combines osteological results, radiocarbon dates on bone apatite, strontium isotopes and concentrations in calcined bones, FTIR-ATR data, and carbon and oxygen isotopes in cremated bones, can open new scenarios in the reconstruction of chronology, human mobility, and funerary practices of the cremated individuals buried at San Valentino in northeastern Italy. Despite the scarce human remains preserved, which prevented an in-depth osteological study, this approach provided new information on this protohistoric key site. New radiocarbon dates allowed to refine the site chronology, which comprises a time span between the end of the Late Bronze Age (10^th^ century BC) and the Early Iron Age (9^th^-7^th^/6^th^ century BC), with an unexpected sporadic occupation in the 4^th^ century BC. Regrettably, the presence of the Hallstatt plateau in the IntCal20 calibration curve did not allow to obtain a more precise date for the site abandonment. A local bioavailable strontium baseline was obtained by analysing samples of modern vegetation collected around the site. The analysis of strontium isotopes in the sampled calcined bones showed no non-local individuals among those buried at San Valentino and analysed in this study; these data, jointly with strontium concentrations measured in the same samples, supported the hypothesis of a local population that exploited local food resources. However, the Sr isotope analyses were not applied to the whole community buried in San Valentino due to the presence of charred (black) bones in several graves, which are not suitable for Sr isotope analysis, and the post-depositional bias responsible for the destruction of part of the graves. The life history of the individual buried in Grave E16 was investigated by measuring strontium isotopes in bone elements with different turnover rates, such as the otic capsule of the petrous portion of the temporal bone, a fragment of diaphysis, and a fragment of cranium. The results showed that the individual was likely born and spent his/her life in the same area where he/she was buried.

FTIR-ATR data showed the presence of cyanamide in 47% of the calcined diaphyses, which could be linked to the body’s position on the funerary pyre that could have led to lower oxygen availability for some of the diaphyses during combustion if the body was in the middle of the pyre instead of on the top based on the study of Salesse et al. [15]. In the case of San Valentino, it seems that there were no temperature-related differences between diaphyses and cranial bones since differences were only observed in IRSF values and not in C/C ratios. The equal burning of the different body parts could be related to the size of the funerary pyre, the amount of wood used during cremation, or the skills of the person(s) who performed the cremation, who made sure that the whole body was homogeneously exposed to fire, ensuring that even the cranium that was at the edges of the funerary pyre was fully burned. The lack of difference in the values of carbon and oxygen isotopes between diaphyses and cranial bones also indicates, like the infrared data, that the whole body was probably burned in a similar way and no differences were detected among the cremated individuals. This indicates that there was likely no variation in the type and/or amount of fuel used for the funerary pyres at San Valentino nor any difference in oxidation conditions during combustion.

The combination of FTIR-ATR data and carbon and oxygen isotopes showed the existence of variations in the cremation conditions (e.g. temperature, pyre structure, location of the pyre in the environment) between San Valentino and the contemporary LBA-EIA urnfield cemeteries of Blicquy, Grand Bois, Herstal, and Velzeke, located in Belgium, which are the only contemporary sites for which these kinds of data are available. The higher δ^13^C values and the greater variability in the range of δ^13^C values observed in San Valentino could be related to the use of different types and/or amounts of wood in the pyre. The differences in δ^18^O values could be related to many factors such as latitude, the position of the body on the pyre, the size, the structure, and the location of the pyre in the environment, or even the season and the amount of wind during cremation. Regarding the infrared results, the cremated bones from San Valentino exhibited lower median IRSF values and C/C ratios compared to the Belgian cemeteries, thus indicating that the burning temperature was generally lower at San Valentino than in the Belgian sites despite the good ventilation conditions suggested by the high δ^18^O values.

## Supporting information

S1 Table Results of ^87^Sr/^86^Sr, [Sr], δ^13^C, δ^18^O, and FTIR-ATR on fragments of calcined human bone from the cremation deposits from San Valentino. (XLSX)
